# Modelling a System to Help Improve It: Comment on "Insights Gained From a Re-analysis of Five Improvement Cases in Healthcare Integrating System Dynamics Into Action Research"

**DOI:** 10.34172/ijhpm.2022.7556

**Published:** 2022-12-04

**Authors:** John Øvretveit

**Affiliations:** ^1^Karolinska Institute, Solna, Sweden.; ^2^Region Stockhom Healthcare, Stockhom, Sweden.

**Keywords:** Action Research, System Modelling, Quality Improvement, Methodology

## Abstract

The article that this commentary considers describes the use of systems modelling in an action research (AR) project that helped improvement teams to understand the dynamics of their service as a system. This commentary seeks to make the complex article easier to understand for those unfamiliar with the subjects. It describes the advantages, disadvantages and benefits, and suggests developments of this approach for research and practice using digital technologies. The conclusion of the commentary is that dynamic system modelling combined with AR is useful for certain purposes and can produce benefits in terms of a more sophisticated understanding of systems and feedback loops for practitioners. However, there are challenges for researchers unfamiliar with AR and dynamic system modelling as well workshop facilitation expertise.

## Introduction

###  What Is the Article About?

 Many readers have some personal experience of an emergency department. The system dynamic (SD) model of the emergency department reproduced in Figure is a good way to begin to understand what the article described by Holmström and colleagues is about.^1^

**Figure:**
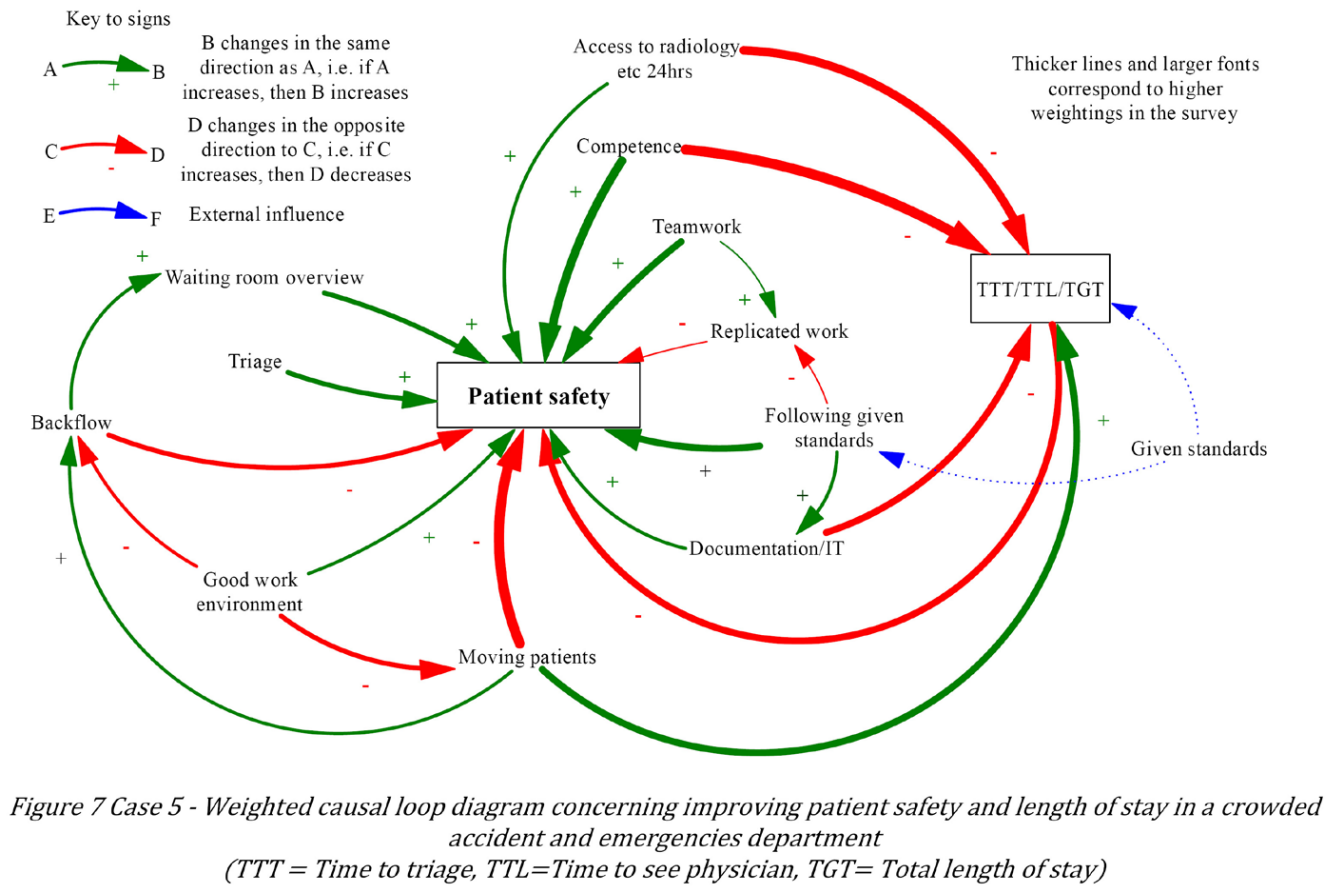
System Model of the Emergency Department (Case 5 in the Appendix to Holmström et al^1^).

 Figure shows the final version of one SD model developed through action research (AR) by the authors of the article for one of five healthcare service delivery cases. This was co-produced by the first author of the article and a project group of emergency department personnel who understood the daily work of the department. The first author of the article served as a facilitator-consultant and worked with the group through 10 stages to produce this model in stage 8. The model is a thus representation of the understanding of the facilitator-consultant and the project group of the causal loops between the key activities of the emergency room that impact both patient safety and the three outcome time-measures of the emergency department operation shown on the right of the Figure. After, and separate to the modelling described in the article, this the clinical project group and others then decided and implemented changes which are not reported.

## Challenges for Readers Unfamiliar With the Subjects

 One aspect of the article that makes it less easy to understand for readers not familiar with these methods is that the article describes the “workflows” of the 10 stages of the project. These are not the workflows of the emergency room, which are the processes usually considered in a quality project. The “testing” described is the extent of agreement of the members of the project team with the representations presented by the facilitator-consultant to the project team, not the real world testing that quality improvement teams often use, with “plan do study act” iterations.^2^ These CQI tests are in reality, not tests of the agreement of group to a representation presented to them.

 In addition, the meaning of “implemented” in the article refers to the model, not to a particular change: the summaries of five cases in table 1 of the article shows that only the obstetrics case as “implemented”: the article uses a classification of degree of implementation as “suggested” (theoretically proposed by the “facilitator-modeller”), “conceptualised” (discussed with a client *organisation), and “implemented” (the model is actually used in practice by the project team). *

 Finally, “simulation” refers to the system feedback loop models, not to other types of simulation used in medical and health services research or in machine learning and artificial intelligence simulation modelling methods.^3,4^ These different meanings of “workflow,” “test,” “simulation,” and “implemented” to the meanings understood by most readers make it more challenging to understand the project presented in the article.

## Advantages, Disadvantages and Value of This SD/AR Approach

 There are benefits to using this approach for certain groups and purposes. For practitioners, which include clinicians, mangers and policy-makers, AR can develop their understanding of the problem and solution-choices during the research, rather than after by reading the report. Their interactive involvement in thinking through the issues engages them in a deeper way. Contributing to solutions can speed implementation as they may choose to make changes earlier, rather than later.^5^ This may also benefit patients and save costs, depending on the objectives of the AR study. As regards using SD modelling in the AR approach described by Holmström et al, benefits to practitioners are to learn about how a system they work with functions, which can reduce the chances of making changes that the model shows are ineffective, or may also have unpredictable negative effects.

 AR provides one approach for some researchers who want their work to impact beneficial change more quickly and directly than occurs in the traditionally steps of research and then dissemination. It also suits researchers who enjoy working closely with practitioners, often in clinical settings. For some researchers with knowledge of SD, combining AR with SD in this way may allow them to collect more valid data directly from those working with the system, and to “test” their model with these practitioners who are more aware of the SD in the later stages of the study.

 Some of the disadvantages and challenges of this SD/AR approach for certain groups and purposes include:

 For practitioners, the stages of the SD/AR approach are time consuming and take them away from other work. For some simple problems, conventional quality improvement, or even everyday problem-solving methods may be less burdensome. My recent experience comparing and AR quality improvement project to implement a large-scale vaccination clinic with a similar project that used simple problem solving by experienced managers found that the simple problem solving was more effective for faster implementation and similar results.^6^

 For researchers not familiar with either SD or AR or without group facilitation process skills, using this specific approach would be challenging. A high level of skills and experience is necessary for credibility with the practitioners and to win their trust in to motivate their willingness to work hard and produce valid data for stages of the study. It is also challenging to both finance such research and to publish it. There are few academic reviewers with knowledge and experience of similar approaches who are qualified to assess proposals for funding and publication manuscripts. Indeed, these are one reason why it is so unusual to see a study of this type carried out or published in a scientific peer reviewed journal. To my knowledge this is the only published detailed study of SD combined with AR. I congratulate the journal for this.

## Discussion: Ways Forward in Dynamic Systems Modelling for Healthcare and Policy Improvement

 New digital technologies could increase the effectiveness and efficiency of this approach and reduce the time taken to carry out such studies. One set of technologies can help to collect data from study participants in a group and analyse and present these data back to the group. Hand-held devices can be used with the group in the physical setting, or data can be collected remotely with a group using real-time Delphi software systems.^7^

 As regards representation and visualization of models, health policy and management research and practice often conceptualizes reality as linear unidirectional causal activities, depicted in a static diagram. Some conceptualizes reality a system or as systems operating at different levels which is difficult to represent. Quality improvement uses systems thinking and methods for testing changes on a small scale, or on small samples, and repeats through iterative improvement steps. Such testing recognizes that system effects are often difficult to predict and allows improvers to abandon a change that is not effective. Most quality improvement does not develop theory about how a particular system operates, as this is not the purpose of practice-based improvement. This, however, is one purpose of research into quality improvement. To produce generalizable knowledge, research using system theory is challenged to discover the precise mechanisms through which many variables affect results.

 SD modelling takes things to a new level of complexity by identifying feedback loops. In the study by Holmström et al, this way of conceptualizing a healthcare service was combined with AR so that clinicians who knew the everyday operations could give researchers feedback about the validity of their model. However, their full understanding of the operation of the feedback loops that the researchers presented in the diagram may be limited. The number of variables and unpredictability about results using such models, in my view, make it even more difficult to understand how the system and its feedback was causing results. A limitation of static diagrams on paper or screen for presenting SD models is that these do not show well how the system operates over time. Static stage-animation using power point and adding parts of the diagram to different slides can help understanding. However, animation software can significantly help clinicians, management and policy-makers to appreciate and understand of the different feedback loops and their cumulative effects on the system over time, as evidenced and described in a recent study.^8^

 “*Process models are widely used for various system analysis and design activities, but it is challenging for stakeholders to understand these complex artifacts. In this work, we focus on the use of dynamic visualization techniques, in particular animation, to help reduce users’ cognitive load when making sense of process models… Our experiments suggested that process model comprehension improves when users of process models are provided with animation features.”*^8^

 Another example is the Gapminder software that presents data visually of trends over time as they unfold.^9^

## Conclusions

 Dynamic system modelling combined with AR is useful for certain purposes and can produce benefits in terms of a more sophisticated understanding of systems and feedback loops for practitioners and develop theory of how systems operate. However, there are challenges for researchers unfamiliar with AR and dynamic system modelling and may not have workshop facilitation expertise. For more complex systems and especially whole systems research and change, the method has many advantages, especially if more use is made of new digital technologies.

## Ethical issues

 Not applicable.

## Competing interests

 Author declares that he has no competing interests.

## Author’s contribution

 JØ is the single author of the paper.
